# 

**Published:** 1826-03

**Authors:** 


### MONTHLY CATALOGUE OF MEDICAL BOOKS.

**** It having been hinted to us that gentlemen, sending copies of their Works, prefer having their titles given at length, it is our intention, in future, to comply with this suggestion: but it is to be observed, that no books can be entered on this List except those sent to us for the purpose;—finding, in the lists hitherto transmitted, that the names of works have frequently been given as published, which have not appeared for weeks, or even months, after.*

Medical and Surgical Cases; selected during a Practice of thirty-nine Years, By Edward Sutleffe, Queen-street, London. Vol. II.—London, 1825.

A Picturesque and Topographical Account of Cheltenham, and its Vicinity. By the Rev. T. D. Fosbroke, M.A. F.A.S. Honorary Associate of the Royal Society of Literature, Honorary Member of the Bristol Philosophical Institution; Author of the Encyclopedia of Antiquities, British Monachism, the History of Gloucestershire, the Wye Tour, &c. To which are added, Contributions towards the Medical Topography, including the Medical History of the Waters. By John Fosbroke, Resident Surgeon at Cheltenham.—Cheltenham, 1826.

### NOTICE TO CORRESPONDENTS.

DR. STOKER's *Paper has been unavoidably postponed till the present Number: we shall be happy to receive the continuation.*

MR. RICHMOND's *Papers in our next.*

MR. OSWALD's *packet has been received.*

☞ *Correspondents are respectfully informed that, when their Communications appear to the Editors not to be consistent with the objects of this Journal, they are left in the hands of the Publisher; by application to whom, they may be obtained.*

